# Alpha-Adrenoceptor Blockade as a Novel Pathway Toward Non-Hormonal Male Contraception: A Mechanistic and Clinical Evidence Review

**DOI:** 10.3390/jcm15176643

**Published:** 2026-08-28

**Authors:** Alicja Roztocka, Patryk Osiński, Halina Car, Natalia Nazarko, Ewa Katarzyna Czech, Emilia Sokołowska

**Affiliations:** 1 Student’s Pharmacological Club, Lazarski University, 02-662 Warsaw, Poland; 18045ckp@lazarski.edu.pl (A.R.); 44452@lazarski.pl (P.O.); 2 Clinical Pharmacology, Medical University of Bialystok, 15-274 Bialystok, Poland; halina.car@umb.edu.pl; 3 Faculty of Law, University of Bialystok, 15-213 Bialystok, Poland; nnazarko@wp.pl (N.N.); e.czech@uwb.edu.pl (E.K.C.)

**Keywords:** alpha-blockers, male contraception, tamsulosin, silodosin, non-hormonal

## Abstract

Alpha-1 adrenergic receptor antagonists are widely used in the management of lower urinary tract symptoms, where they can induce ejaculatory dysfunction as a pharmacodynamic effect, particularly with α1A-selective agents. This observation has led to growing interest in their potential role as non-hormonal male contraceptives. This narrative review evaluates current clinical and preclinical evidence regarding the impact of α-blockers on the ejaculatory mechanism, with a focus on their potential application in male contraception. Both human and animal data were examined to contextualize the mechanistic basis for contraceptive potential. Evidence from clinical and pharmacological studies indicates that inhibition of α1-adrenoceptor-mediated smooth-muscle contraction in tissues involved in seminal emission, including the vas deferens, seminal vesicles, and prostate, can impair the emission phase of ejaculation. Agents such as tamsulosin and silodosin have been reported to induce anejaculation or markedly reduce semen volume, providing pharmacodynamic evidence of impaired seminal emission; however, these effects should not be considered equivalent to demonstrated contraceptive efficacy. Prospective clinical evidence currently includes one prospective pilot study and one randomized placebo-controlled clinical trial evaluating silodosin as a potential reversible non-hormonal male contraceptive. The prospective evidence includes marked and progressive suppression of sperm output and semen volume with repeated silodosin administration; at week 12, 94% of participants achieved a total sperm count ≤ 1 × 10^6^ per ejaculate, while the observed pregnancy incidence was 4% versus 17% with placebo. However, the available evidence remains insufficient to establish reliable contraceptive effectiveness for individual sexual exposures. Safety considerations, including cardiovascular effects and sexual dysfunction, also remain insufficiently characterized in healthy men. α1-adrenoceptor antagonists represent a biologically plausible pharmacologic strategy for male non-hormonal contraception by interfering with seminal emission and sperm delivery without directly suppressing spermatogenesis. Further pharmacokinetic–pharmacodynamic studies and rigorously designed multicentre clinical trials are required to determine the optimal dosing strategy, completeness and consistency of seminal emission suppression, contraceptive effectiveness, safety, acceptability, and reversibility of this approach.

## 1. Introduction

Unintended pregnancy remains a significant public health concern worldwide, notwithstanding considerable progress in contraceptive technology [1,2]. Current male contraceptive options remain limited largely to condoms and vasectomy, while several pharmacological approaches are still under clinical development. This unmet need has stimulated increasing interest in reversible non-hormonal methods that could prevent sperm delivery without altering endocrine function [1,2,3,4].

Currently, condoms remain the only widely used reversible male contraceptive method, whereas vasectomy is considered a highly effective but intended-to-be permanent method of contraception, despite the possibility of surgical reversal [5]. Condoms are non-invasive but have a failure rate of approximately 13%. Vasectomy, in contrast, is a surgical intervention involving the cutting and sealing of the vas deferens. It is a highly effective procedure (less than 1% failure rate), typically performed under local anesthesia [6,7]. In comparison, women have access to a broader and generally more reliable range of contraceptive methods, including hormonal intrauterine systems, combined oral contraceptives, hormonal implants, injections, and copper Intrauterine Devices (IUDs) [8]. A multinational survey conducted by Heinemann et al. [9] among 9000 men aged 18–50 in nine countries revealed that roughly half of the respondents would be willing to use a reversible male contraceptive method if such an option were available. More recent international evidence has demonstrated sustained interest in novel male contraceptive methods, while showing that willingness to use such methods depends not only on efficacy and reversibility but also on safety, convenience, and other product attributes [10].

To date, hormonal male contraceptives represent the most advanced area of clinical development. However, achieving an optimal balance between reliable suppression of spermatogenesis, maintenance of physiological androgen activity, safety, user acceptability, and the development of an effective oral formulation remains a major challenge [11].

In parallel, non-hormonal male contraceptive strategies are being investigated as an alternative approach to preventing fertilization without directly suppressing the hypothalamic–pituitary–gonadal axis [12,13,14,15]. These strategies may target different stages of male reproductive function, including spermatogenesis, sperm maturation and function, or sperm transport and seminal emission. Approaches directed at sperm transport or seminal emission are of particular interest because they may interfere with the delivery of sperm without directly impairing sperm production or systemic hormonal function. They may also offer a more rapid onset of contraceptive action than approaches that require suppression of spermatogenesis. However, the extent to which these pharmacodynamic effects can be translated into reliable pregnancy prevention remains to be established.

Within this broader group of non-hormonal approaches, α1-adrenoceptor antagonists have attracted particular interest because their established pharmacological effects on smooth-muscle contraction extend to tissues involved in seminal emission. These medications are primarily used to treat lower urinary tract symptoms (LUTS) associated with benign prostatic hyperplasia (BPH) [16]. Their potential contraceptive effect is based on inhibition of α1-adrenoceptor-mediated smooth-muscle contraction in the reproductive tract, including the vas deferens, seminal vesicles, and prostate, thereby impairing the emission and delivery of seminal contents during ejaculation. Three α1-adrenoceptor subtypes have been identified: α1A, α1B, and α1D [17]. The α1A subtype predominates in the lower urinary tract [18], while α1B is more closely associated with vascular tone regulation [19]. Thus, agents with high α1A selectivity and low α1B affinity may relieve urinary symptoms while minimizing cardiovascular adverse effects. Among these, tamsulosin is widely prescribed for urinary disorders, while silodosin demonstrates higher α1A selectivity and a lower propensity for cardiovascular adverse effects [20]. This pharmacological profile provides a rationale for investigating selective α1A-adrenoceptor blockade as a potential non-hormonal contraceptive strategy, while also raising an important development question: whether inhibition of seminal emission can be made sufficiently complete and consistent to provide reliable protection against pregnancy.

**Figure 1:**
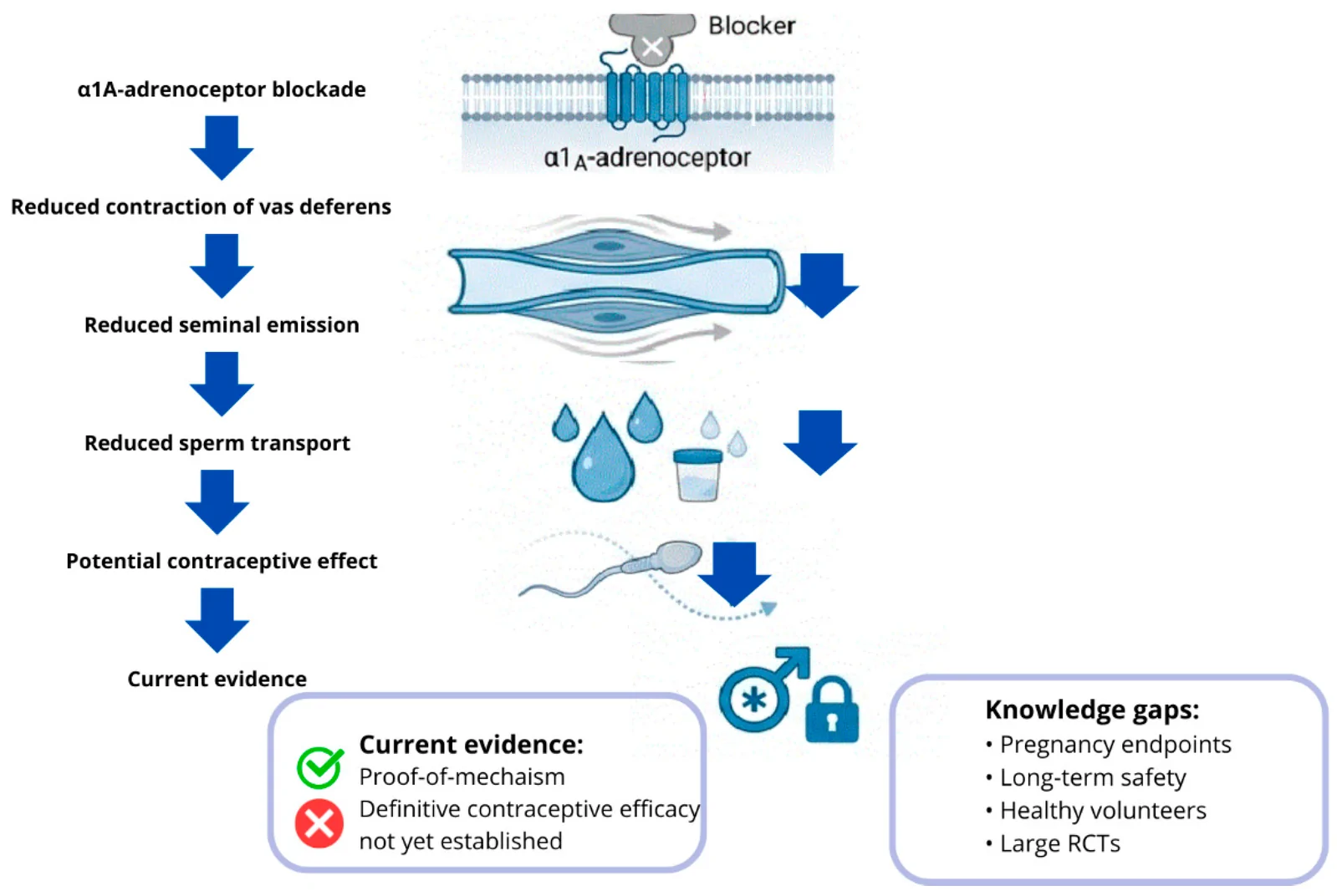
Proposed mechanism of α1A-adrenoceptor blockade as a potential non-hormonal male contraceptive. Current evidence supports biological plausibility and early clinical proof-of-concept, including prospective evidence of marked suppression of sperm delivery and a lower observed pregnancy incidence; however, reliable contraceptive effectiveness has not yet been established.

Unlike hormonal contraceptive approaches, α1-adrenoceptor antagonists do not directly suppress spermatogenesis or alter the hypothalamic–pituitary–gonadal axis. Instead, their proposed contraceptive effect involves interference with the emission phase of ejaculation through inhibition of α1A-adrenoceptor-mediated smooth-muscle contraction in the vas deferens, seminal vesicles, and prostate. Consequently, the proposed contraceptive effect is based on impaired sperm transport and seminal emission rather than impaired sperm production (Figure 1). Importantly, suppression of sperm delivery represents a pharmacodynamic mechanism and should not automatically be equated with contraceptive effectiveness. Reliable contraceptive development requires demonstration that the intervention prevents pregnancy in appropriately designed clinical studies.

Because men do not face the medical risks associated with pregnancy and childbirth, determining an acceptable balance between benefits and potential harms of a male contraceptive tends to be particularly challenging. Accordingly, contraceptive agents intended for men would require a particularly favorable balance between efficacy, safety, tolerability, and acceptability. In practice, this means that several years of clinical testing, often involving large numbers of couples, are required before any such product could realistically reach the market. Until now, the FDA has not approved a male contraceptive pill. However, regulatory development of a repurposed α1-adrenoceptor antagonist would build on an existing body of pharmacokinetic, pharmacological, manufacturing, and safety information, while still requiring new evidence appropriate to the proposed contraceptive indication [21]. Regulatory considerations relevant to repurposing are discussed separately below.

The clinical acceptability of an emission-blocking contraceptive also warrants specific consideration because the intended pharmacological effect may be experienced by users as a change in ejaculation. Kaur et al. [10] specifically incorporated ejaculation-related changes, including reduced or absent ejaculate, among the attributes evaluated in a large multinational discrete-choice study of potential male contraceptive users and their female partners. The study indicated that administration characteristics, including the timing and mode of use, were among the strongest determinants of product preferences, whereas ejaculation-related changes had a smaller relative influence on decision-making. However, hypothetical preference data cannot establish the acceptability of drug-induced changes in ejaculation in men actually receiving an emission-blocking contraceptive, and this question requires dedicated clinical evaluation.

In this narrative review, we first briefly discuss the physiological mechanism of ejaculation and the potential for pharmacological modification. Using data from clinical trials, we then critically review the current evidence regarding α1-adrenoceptor antagonists as candidates for reversible male contraception. Particular attention is given to the distinction between pharmacodynamic suppression of seminal emission and demonstrated contraceptive effectiveness, as well as to the pharmacokinetic–pharmacodynamic considerations underlying different dosing strategies. We also assess the safety, acceptability, reversibility, and regulatory considerations relevant to further development of α1-adrenoceptor antagonists as male contraceptives.

## 2. Search Strategy

This narrative review was conducted to synthesize mechanistic, preclinical, and clinical evidence regarding the effects of α-adrenergic receptor antagonists on ejaculation and their potential application in non-hormonal male contraception. The literature search was designed to identify relevant mechanistic, preclinical and clinical studies and was not intended to constitute a formal systematic review or meta-analysis. The search strategy was developed to identify clinical trials, observational studies, and preclinical studies on the effects of α-adrenergic receptor antagonists on ejaculatory function, male fertility, and their potential use as non-hormonal contraceptives. The search was conducted in the ClinicalTrials.gov, PubMed, Scopus, and Web of Science databases, covering publications without date restrictions. Searches were updated through May 2026. Only English-language publications were included. Clinical trial registries were searched irrespective of study status, including “completed,” “active,” “terminated,” and “withdrawn” studies. The search process included predefined keywords and controlled vocabulary where applicable, followed by screening of reference lists and cited literature to identify additional relevant studies.

## 3. Mechanistic Insights into Sympathetic-Driven Seminal Emission and α1-Adrenoceptor Blockade

α1A-adrenoceptors are not exclusively involved in reproductive function. Their established therapeutic role in male lower urinary tract symptoms is based on relaxation of α1A-mediated smooth-muscle tone in the prostate, bladder neck and urethra, thereby reducing dynamic outlet resistance. The same α1A-adrenoceptor-dependent contractile signaling that contributes to bladder-neck and prostatic smooth-muscle tone also contributes to contraction of reproductive smooth muscle during seminal emission. During ejaculation, sympathetic α1-adrenoceptor activation coordinates contraction of the vas deferens, seminal vesicles and prostate, facilitating movement of sperm and seminal fluid toward the prostatic urethra. Pharmacological blockade of these receptors can therefore impair the emission phase without necessarily abolishing orgasmic sensation.

Thus, the proposed contraceptive effect of selective α1A-adrenoceptor antagonists is not a separate pharmacological action but an extension of their established smooth-muscle pharmacology to reproductive tissues. Importantly, impaired seminal emission should be distinguished from retrograde ejaculation. Available human mechanistic studies with silodosin indicate that the predominant mechanism appears to involve inhibition of seminal emission rather than obligatory retrograde ejaculation.

Ejaculation consists of two stages: emission and expulsion [22]. During this process, the smooth muscles of the seminal vesicles, vas deferens, prostate, and bladder neck contract. For the penile venous muscles to relax, intracellular calcium levels must decrease, with neurotransmitters such as nitric oxide (NO) and cyclic guanosine monophosphate (cGMP) playing a crucial role [23]. Studies examining pressures within the seminal vesicles and vas deferens have shown that the administration of selective adrenergic receptor antagonists suppresses contractions in these regions of the rat reproductive tract [24].

Proper ejaculation results from the interaction of the sympathetic, parasympathetic, and somatic nervous systems, with the sympathetic system playing the dominant role. It releases norepinephrine [22], which activates α1-adrenoceptors [25], leading to contraction of the vas deferens, seminal vesicles, and prostate [26], as well as the smooth muscle of the cauda epididymis [27]. The use of α1-adrenoceptor antagonists appears to reduce sperm delivery during ejaculation [28], forming the biological rationale for exploring their potential as non-hormonal contraceptive agents [29].

As noted above, smooth muscle relaxation requires a reduction in intracellular calcium levels, which can be achieved by blocking α1-adrenoceptors. The contraction mechanism involves norepinephrine and epinephrine binding to α1-adrenoceptors coupled to G-protein. This activates phospholipase C and downstream phosphorylation events, resulting in the production of secondary messengers inositol triphosphate (IP3) and diacylglycerol (DAG). The final outcome is an increase in intracellular Ca^2+^ and subsequent muscle contraction [30].

The emission phase involves the release of sperm and other seminal components from the accessory genital glands. This includes peristaltic contractions of the vas deferens, which propel sperm toward the urethra [31].

A study by Kimura et al. [32] elucidated the α-adrenergic mechanisms underlying sperm emission and ejaculation. In rats, α-adrenergic antagonists were shown to reduce seminal emission induced by hypogastric nerve stimulation and to abolish rhythmic contractions of the posterior urethra. In contrast, α-agonists induced contractions, while β-agonists and β-blockers had no significant effects. Additionally, the P2X1 purinoceptor is known to contribute to the ejaculatory process, primarily by regulating sperm transport through the vas deferens during ejaculation [33]. Deletion of P2X1 receptors caused infertility in 86% of mice, whereas neither spermatogenesis nor sexual behavior was altered in mice lacking both α1A and P2X1 receptors [29].

Supraspinal centers also play an essential role in controlling the spinal ejaculation generator. These include structures involved in regulating sexual behavior, such as the medial preoptic area (MPOA), paraventricular nucleus of the hypothalamus (PVN), and nucleus paragigantocellularis (nPGi). The MPOA is critical for initiating and regulating sexual behavior [31]; its damage results in inhibition of ejaculation in rodents. Neurons in the nPGi show serotonergic activity, and Marson et al. [34] demonstrated that serotonin administration into the spinal cord inhibits ejaculation. Thus, serotonin acts as an inhibitory modulator of the ejaculatory reflex [35]. Ejaculatory delay is mediated through activation of spinal and supraspinal 5-HT1B and 5-HT2C receptors [36]. Kim and Paick [37] examined the effects of serotonin on ejaculation in rats and found higher mRNA expression of 5-HT1B and 5-HT2C receptors in the seminal vesicles than in the vas deferens.

Ahlenius and Larsson [38] demonstrated that activating 5-HT1B receptors inhibits ejaculation. In subsequent studies [39], they examined the effects of N-[2-[4-(2-methoxyphenyl)-1-piperazinyl]ethyl]-N-(2-pyridinyl)cyclohexanecarboxamide 3HCl (WAY-100635) and citalopram, both 5-HT1A receptor antagonists. Citalopram had no significant effect, while WAY-100635 increased the number of mounts and reduced the number of intromissions. The 5-HT1B agonist isamoltane antagonized these effects. Combined treatment with higher doses of both agents delayed ejaculation, increased the number of pre-ejaculatory mounts, and prolonged post-ejaculatory intervals. These findings suggest that 5-HT1A receptor blockade reveals an inhibitory effect of citalopram on ejaculation mediated through 5-HT1B receptor activation.

Finally, Ahlenius et al. [40] investigated various serotonergic compounds. The 5-HT agonist 8-methoxy-2-(di-n-propylamino)tetralin (8-OH-DPAT) at a dose of 16 mg/kg increased ejaculatory latency and reduced sexual behavior. Conversely, 5-hydroxy-2-(di-n-propylamino)tetralin (5-OH-DPAT) at 0.4 mg/kg both decreased sexual behavior and shortened ejaculatory latency.

## 4. Preclinical Animal Research Supporting α1-Adrenoceptors Antagonists as Potential Male Contraceptives

Ratnasooriya W.D. et al. [41] investigated the effects of tamsulosin on infertility. The study was conducted in rats administered tamsulosin at a dose of 0.15 mg/kg or with an equivalent volume of vehicle between 3 p.m. and 4 p.m. The following parameters were evaluated: sexual behaviour, libido index, vaginal sperm count, number of implants, pregnancy rate, fertility index, pre-implantation loss, and post-implantation loss. All values were assessed seven days before the study, on the day of tamsulosin administration, and three and seven days after drug administration. Treatment was associated with a decrease in vaginal sperm count (day 1 = 0.45 × 10^6^, day 3 = 5.54 × 10^6^), and with an azoospermia (16.6% and 33.3% respectively). A reduction in the number of uterine implants and an increase in pre-implantation loss were observed after days 1 and 3 of treatment. However, no effect on post-implantation loss was noted. Pregnancy rates and fertility decreased significantly only after the third day of the trial. Subsequently, Tatemichi et al. [42] administered tamsulosin (0.1 mg/kg), silodosin (1 mg/kg), alfuzosin (3 mg/kg), and naftopidil (30 mg/kg), drugs with inhibitory effects on α1-adrenoceptors in the lower urinary tract involved in ejaculation, to rats per os (po). Treatment resulted in a reduction in the number of copulatory plugs and an increase in seminal vesicles weight. Tamsulosin was the most potent inhibitor, followed by silodosin, alfuzosin and naftopidil. The authors suggested that abnormal ejaculation may be a consequence of inhibition of seminal vesicle and seminal vesicle contractions.

Other fertility studies have investigated the effects of tamsulosin and silodosin on the seminal vesicles of guinea pigs. These studies demonstrated a clear inhibitory effect of both drugs, as electrically induced contractions were significantly reduced following their administration. Hayashi et al. [43] suggested that α_1_-adrenergic receptor antagonists primarily affect the emission phase of ejaculation. Tambaro et al. [44] also suggested that tamsulosin inhibits vas deferens contractions, whereas alfuzosin did not abolish contractile activity. This difference may be attributable to alfuzosin’s lower receptor affinity or reduced absorption into the vasculature. Consistent with these findings, Giuliano et al. [24] reported a higher incidence of ejaculatory dysfunction with tamsulosin (4–18%) compared with alfuzosin (<1%).

Similarly, Kim et al. [45], in a study conducted in rats treated with prazosin, terazosin, and tamsulosin, demonstrated that these drugs reduced pressure in the seminal vesicles and vas deferens. At a dose of 10 mg/kg, all three agents exerted a greater effect on pressure changes in the seminal vesicles than in the vas deferens. Earlier work by Ratnasooriya et al. [46] described a study in which a constricting collar was placed around the seminal duct. A decrease in fertility was observed, accompanied by an increased incidence of hypozoospermia. Spermatozoa in the ejaculate appeared immotile, and some exhibited decapitation. No significant effect of prazosin on mating behaviour, as assessed by the copulation index, was observed. These findings suggest that prazosin primarily interferes with ejaculation rather than spermatogenesis. This hypothesis is supported by the observation that no antisepermatogenic effect was detected during the first weeks of collar treatment [41]. Solomon et al. [28] conducted a study to further substantiate this hypothesis. In an investigation of sperm transport in male rats, spermatozoa collected from the cauda epididymis showed no changes in motility or total sperm count. However, seminal fluid collected after ejaculation contained fewer spermatozoa. The distal epididymis of treated rats contained 89% fewer spermatozoa than that of the control group. In controls, 93% of spermatozoa were expelled during ejaculation, with the remainder retained in the distal epididymis, whereas treated rats expelled only 65%. Prazosin is an α_1_-adrenergic receptor antagonist and, through its blocking action, is presumed to inhibit smooth muscle contraction of the seminal vesicles and vas deferens. Consequently, prazosin impairs the transport and secretion of seminal fluid.

In summary, animal in vivo studies show that α1-adrenoceptor antagonists such as tamsulosin, silodosin, prazosin, and terazosin can impair the emission phase of ejaculation by inhibiting smooth-muscle contractions in the seminal vesicles and vas deferens. These drugs reduce sperm expulsion, lower vaginal sperm counts, and can induce transient azoospermia, leading to decreased implantation rates and reduced fertility in treated animals. Tamsulosin appears to exert the strongest inhibitory effect, whereas alfuzosin shows minimal influence, likely due to lower receptor affinity or tissue penetration. Importantly, sperm production and motility generally remain intact, indicating that reduced fertility results primarily from impaired seminal transport rather than direct gonadotoxicity. Together, these findings support further investigation of α-blockers as mechanistically plausible candidates for male contraceptive development. Although animal studies provide important mechanistic evidence, their findings cannot be directly extrapolated to human contraceptive efficacy owing to species-specific differences in reproductive physiology.

## 5. Clinical Trial Evidence on the Contraceptive Potential of α1-Adrenoceptor Antagonists

Some α1-adrenergic receptor antagonists, particularly those with high affinity for α1A receptors, are associated with ejaculatory dysfunction. These drugs can interfere with the function of the vas deferens, resulting in impaired seminal emission and potentially reduced fertility [47].

Analysis of available data from the ClinicalTrials.gov registry and a review of the literature indicates that although α1-adrenergic blockers are a well-studied group of drugs in the context of lower urinary tract disorders and benign prostatic hyperplasia (NCT01404637), their effects on ejaculatory function are well documented and provide a rationale for further investigation as potential contraceptive agents (Table 1). In most of the identified clinical trials with tamsulosin, silodosin or alfuzosin, ejaculation disorders were recorded as expected or common adverse events, supporting an effect of α1-adrenergic receptor blockade on the emission phase of ejaculation. Although formal semen analyses were rarely the primary endpoint, data from protocols and published results describe a reduction in ejaculate volume and, in many cases, the phenomenon of ‘dry orgasms’, i.e., the absence of visible ejaculation despite a preserved sexual response. A particularly strong effect is observed in the case of drugs with high selectivity for the α1A subtype, which is dominant in the structures responsible for sperm transport.

Studies involving tamsulosin and silodosin indicate that a reduction in ejaculate volume or its absence occurs in a significant percentage of patients, especially in younger populations, which was also observed in protocols for the treatment of premature ejaculation (PE), where the mechanism of emission blockage was one of the components of the therapeutic effect (NCT03879746). The observed side effects are consistent with a mechanism that could be potentially exploited to develop pharmacological, reversible inhibition of semen emission without disrupting spermatogenesis and the endocrine system. Data from the study registry, including studies on tamsulosin, prazosin, alfuzosin and drug combinations, repeatedly indicate that ejaculation disorders are a reproducible pharmacological effect, predictable based on the pharmacodynamics of the drug and dependent on the dose and receptor selectivity.

A prospective, randomized, double-blind, placebo-controlled study by Nouh et al. [48] evaluated 200 healthy, sexually active, fertile men, with 100 participants receiving silodosin 8 mg and 100 receiving placebo. The study used a continuous treatment framework, with silodosin administration scheduled at night or approximately 3 h before intercourse when applicable. Adherence was monitored using intercourse diaries and pill counts [48]. Total spermatozoa per ejaculate, semen volume, and post-ejaculatory urinalysis were assessed after 2, 6, and 12 weeks.

The effect on sperm output was progressive over the study period. The median total spermatozoa per ejaculate decreased from 27.95 × 10^6^ at week 2 to 11.7 × 10^6^ at week 6 and 0.0005 × 10^6^ at week 12, whereas corresponding placebo values remained approximately 76–77 × 10^6^. All between-group comparisons were statistically significant (*p* < 0.001), and within the silodosin group each successive time point was significantly lower than the preceding one [48]. At week 12, 94% of participants had a total sperm count of ≤1 × 10^6^ per ejaculate. Semen volume showed a parallel progressive reduction, from a median of 1.495 mL at week 2 to 1.15 mL at week 6 and 0.605 mL at week 12, compared with approximately 2.4 mL in the placebo group [48]. Importantly, the marked reduction in sperm output was not accompanied by complete anejaculation in all treated participants; at week 12, the reported semen volume was 0.605 mL (0.55–0.64 mL), indicating profound hypospermia rather than uniform absence of ejaculate.

Post-ejaculatory urinalysis provided additional evidence of progressive suppression of sperm delivery: spermatozoa were detected in 81%, 43%, and 10% of participants in the silodosin group at weeks 2, 6, and 12, respectively, compared with 100% of placebo-treated participants at each time point [48]. However, the absence of spermatozoa in post-ejaculatory urine should not be equated automatically with complete inhibition of seminal emission or azoospermia in the ejaculate. Importantly, the marked reduction in total sperm count observed in this study should likewise not be interpreted as equivalent to contraceptive efficacy, because a validated surrogate endpoint for prevention of pregnancy has not been established for pharmacological blockade of seminal emission.

Pregnancy occurred in 4% of participants in the silodosin group compared with 17% in the placebo group (χ^2^ = 36.291, *p* < 0.001) [48]. The randomized placebo-controlled design, prospective pregnancy assessment, exclusion of concomitant contraceptive methods, and monitoring of intercourse and dosing adherence make this finding clinically relevant; however, the pregnancy data cannot by themselves establish contraceptive efficacy. Nevertheless, the study does not establish contraceptive effectiveness for individual sexual exposures. The publication does not provide sufficient information on the number of intercourse events or couple-months of exposure, the timing of individual pregnancies, or whether conception occurred following fully protocol-adherent dosing. Furthermore, baseline semen parameters were not reported, limiting interpretation of the magnitude of within-subject change from pretreatment values.

The progressive decline observed with repeated administration should also not be interpreted as evidence of drug accumulation. Although the authors proposed a possible cumulative effect, they acknowledged that further clinical and pharmacological studies are required to determine the mechanism underlying the progressive suppression [48]. Thus, the available data demonstrate a progressive pharmacodynamic effect during repeated treatment, but do not establish the mechanism responsible for this time-dependent increase in suppression.

These findings should also be interpreted in the context of the first prospective clinical study specifically designed to evaluate the contraceptive potential of an α1A-adrenoceptor antagonist, conducted by Bhat and Shastry [49] and published in 2020. Unlike previous studies, in which ejaculatory dysfunction was reported only as an adverse event during treatment of benign prostatic hyperplasia, this pilot trial directly investigated whether silodosin could be used as an on-demand, reversible, non-hormonal male contraceptive. In that study, 63 healthy fertile men received silodosin 8 mg approximately 3 h before heterosexual intercourse during the contraceptive phase. Reversible azoospermia was demonstrated during the crossover phase, and no unintended pregnancies were reported during the subsequent one-year follow-up; four participants were lost to follow-up [49]. Despite the encouraging results, the findings should be interpreted with caution. This was a pilot study with a relatively small sample size, conducted at a single center, and its results have not yet been independently replicated. Unlike the daily regimen evaluated by Nouh et al. [48], the Bhat and Shastry study therefore directly investigated an on-demand dosing strategy linked temporally to sexual intercourse.

Taken together, these studies provide complementary but not interchangeable evidence. Bhat and Shastry [49] provide preliminary evidence that administration approximately 3 h before intercourse can produce reversible azoospermia in the studied participants, whereas Nouh et al. demonstrate that repeated daily administration produces profound suppression of sperm output but does not eliminate pregnancy occurrence. These approaches should not be considered interchangeable. Bhat and Shastry [49] evaluated temporally targeted administration before intercourse, whereas Nouh et al. [48] evaluated repeated treatment over 12 weeks. The progressive suppression observed during repeated administration in the Nouh et al. [48] study raises the possibility that repeated treatment may produce a pharmacodynamic effect beyond the acute inhibition of seminal emission observed after administration shortly before intercourse. However, this remains a hypothesis and cannot be attributed to drug accumulation on the basis of the available clinical data. Conversely, the results of Bhat and Shastry [49] cannot be extrapolated to continuous 24 h contraceptive protection.

Given the reported pharmacokinetic profile of silodosin, including a Tmax of approximately 2.5 h and a half-life of approximately 13 h, the repeated 8 mg regimen cannot be assumed to provide continuous pharmacological coverage of seminal emission throughout the dosing interval. Direct pharmacokinetic–pharmacodynamic studies will therefore be required to establish whether effective contraceptive use is best achieved through temporally targeted administration or sustained exposure. This creates two potentially distinct development pathways: optimization of a rapid-onset formulation for reliable on-demand use or development of a formulation and dosing regimen capable of maintaining sufficient pharmacodynamic coverage for repeated daily use. The difference between these dosing strategies highlights an important unresolved pharmacological question: whether reliable contraceptive protection depends primarily on achieving a sufficiently high exposure at the time of seminal emission or on maintaining pharmacologically effective exposure throughout the dosing interval. This question cannot be resolved from the currently available clinical data and requires dedicated pharmacokinetic–pharmacodynamic studies linked to standardized semen and reproductive outcomes.

Thus, the results of a review of the literature and clinical trial registry suggest that α1-blockers may provide a biologically plausible conceptual basis for the development of a new class of male contraceptives that act primarily at the level of seminal emission and sperm transport. Their action appears to be reversible and quickly disappears after discontinuation of treatment, without affecting the male hormonal balance, which is a potential advantage over the methods developed so far. At the same time, dedicated clinical trials specifically evaluating pregnancy prevention, contraceptive reliability and long-term acceptability remain limited.

Clinical studies suggested that selective α1A-adrenoceptor antagonists can induce anejaculation or markedly reduce semen volume. However, most available evidence originates from trials conducted in patients with benign prostatic hyperplasia or lower urinary tract symptoms. In these studies, ejaculatory dysfunction was reported as an adverse event rather than a predefined contraceptive endpoint. Therefore, the current body of evidence has progressed beyond proof-of-mechanism and now represents early clinical proof-of-concept rather than definitive proof of contraceptive efficacy. These findings support continued clinical translation of selective α1A-adrenoceptor blockade while emphasizing the need for confirmatory multicentre trials before routine clinical application can be considered.

Beyond studies evaluating currently approved α1-adrenoceptor antagonists, recent years have witnessed the emergence of dedicated clinical development programs focused specifically on non-hormonal male contraception. This represents an important shift from retrospective observations in patients treated for lower urinary tract symptoms toward prospective investigations designed to evaluate reproductive outcomes. Building upon the encouraging findings of the pilot study by Bhat and Shastry [49] and the subsequent randomized placebo-controlled evaluation of silodosin in healthy fertile men [48], research has now progressed toward purpose-designed contraceptive drug development.

Further evidence of continuing clinical development is provided by the ongoing Phase IIa clinical trial NCT07393334 evaluating the investigational compound NLS-133 in healthy male volunteers. Unlike previous studies performed in patients with LUTS/BPH, this trial has been specifically designed to assess reproductive outcomes, including semen volume, sperm count, sperm motility, and ejaculation quality following single-dose administration. As no results have yet been published, the study should currently be regarded as an important step in clinical development rather than evidence of contraceptive efficacy.

The transition from experimental and clinical investigation toward potential product development is also reflected by patent activity. Patents including US10912762B2 and EP3562485B1 describe non-hormonal male contraceptive compositions and methods based on (R)-silodosin, including daily oral administration and modified-release formulations intended to optimize drug exposure. However, patent protection should not be considered evidence of clinical efficacy or regulatory approval; the contraceptive potential of these approaches ultimately requires confirmation in well-designed clinical trials.

Taken together, the currently available evidence illustrates the progressive clinical development of α1-adrenoceptor antagonists from incidental observations of ejaculatory dysfunction to dedicated prospective contraceptive studies and purpose-designed drug-development programmes. Although the available clinical evidence has expanded considerably, it remains insufficient to support routine clinical application. Large, multicentre prospective trials with pregnancy prevention as the primary endpoint, longer follow-up, and independent validation are still required before α1-adrenoceptor antagonists can be considered clinically applicable non-hormonal contraceptive agents.

**Table 1:** Clinical trials involving α1-adrenoceptor blockers and ejaculatory or fertility outcomes.

Study Title/Identifier (Status)	Study Type	Drug/Intervention	Ejaculation or Semen Analysis Mentioned?	Notes/Source
Effects of Tamsulosin 0.4 mg on Clinical Outcomes in Korean Men With Severe Symptomatic Benign Prostatic Hyperplasia (BPH) Identifier: NCT01404637 (completed) Ref. [50].	Interventional, Randomized, Parallel Assignment, Single-blinded	Experimental: Tamsulosin 0.4 mg Active Comparator: tamsulosin 0.2 mg	Yes–ejaculatory function assessed; The incidence of adverse events: abnormal ejaculation 1.8%	Ejaculatory effects described in associated studies
Effect of Tamsulosin on PE Compared With Paroxetine Hydrochloride. Identifier: NCT03879746 (completed)	Interventional, Single Group Assignment, Open Label	Active Comparator: tamsulosin 0.4 mg	Not directly; ejaculatory AEs known	PE study; ejaculatory changes described
Actual Use Study of Tamsulosin in Men Identifier: NCT02573311 (completed) Ref. [51].	Interventional, Randomized, Factorial Assignment, Open Label	Active Comparator: tamsulosin 0.4 mg	Yes (AEs related to ejaculation). The incidence of adverse events: ejaculation disorder (1.7%), semen volume decrease (1.7%)	Real-world safety monitoring includes sexual AEs
Comparison of Silodosin and Tamsulosin Identifier: for Medical Expulsive Therapy in Patients With Ureteral Stones Identifier: NCT06999135 (completed)	Interventional, Randomized, Parallel Assignment, Open Label	Active Comparator: tamsulosin, Drug: Silodosin; Experimental: Silodosin, Drug: Silodosin	Registry record exists—safety profile comparison included; ejaculatory AE frequencies not reported in the public summary.	Ejaculation mentioned as AE
Deprescribing Tamsulosin in Older Men (PERSONAL) Identifier: NCT05415748 (completed)	Interventional, Randomized, Crossover Assignment, Quadruple-blinded	Experimental: Tamsulosin 0.4 mg or 0.8 mg,	Yes (sexual AEs tracked)	Focus on withdrawal outcomes in older men
A Study to Evaluate the Efficacy, Safety, and Tolerability of Mirabegron in Men With OAB Symptoms While Taking Tamsulosin Hydrochloride for Lower Urinary Tract Symptoms (LUTS) Due to Benign Prostatic Hyperplasia (BPH) (PLUS) Identifier: NCT02757768 (completed) Ref. [52].	Interventional, Randomized, Parallel Assignment, Double-blinded	Tamsulosin 0.4 mg (oral tablet) throughout the study. Mirabegron: 25 mg of mirabegron (oral tablet) which was increased to 50 mg after 4 weeks	Partially	Sexual AEs sometimes included
A prospective, double-blind, randomized, placebo-controlled study to evaluate the efficacy of silodosin 8 mg as an on-demand reversible non-hormonal oral contraceptive for males: A pilot study Identifier: CTRI/2017/09/009872 (completed) Ref. [49].	Prospective, randomized, double-blind, placebo-controlled pilot study	Silodosin 8 mg administered approximately 3 h before intercourse	Yes. Semen analysis demonstrated reversible azoospermia. Pregnancy outcomes and treatment safety were also assessed.	Pregnancy outcomes and treatment safety were also assessed. First prospective clinical study specifically designed to evaluate the contraceptive potential of an α1A-adrenoceptor antagonist. No unintended pregnancies were reported during the one-year follow-up; however, findings require confirmation in larger studies.
Silodosin as a Male Contraceptive Non Hormonal Identifier: NCT07195097 (completed) Ref. [48].	Interventional, Randomized, Parallel Assignment, Placebo-Controlled Clinical Trial	Silodosin 8 mg; continuous treatment with dosing scheduled at night or approximately 3 h before intercourse when applicable	Primary outcome: Pregnancy rate; Secondary outcomes: total sperm count per ejaculate, semen volume, post-ejaculatory urine analysis	Primary outcome: Pregnancy rate; Secondary outcomes: total sperm count per ejaculate, semen volume, post-ejaculatory urine analysis Condition: Contraception Use. A total of 200 healthy, sexually active, fertile men were randomized (100 silodosin; 100 placebo). At week 12, median total spermatozoa/ejaculate 0.0005 × 10^6^ vs. 77.10 × 10^6^; 94% achieved ≤1 × 10^6^/ejaculate; median semen volume 0.605 vs. 2.4 mL; pregnancy incidence 4% vs. 17%.
A Study to Evaluate NLS-133 in Healthy Male Volunteers (NCT07393334)	Phase IIa, randomized, quadruple-blind, placebo- and active-controlled crossover study	Single oral dose of NLS-133 administered 90 or 180 min before semen collection	Yes. Primary endpoints include semen volume and sperm count; secondary endpoints include sperm motility, morphology, ejaculation quality, orgasm quality, pharmacokinetics, and safety.	Ongoing clinical trial evaluating an investigational on-demand non-hormonal male contraceptive in healthy volunteers. No efficacy results are currently available.

AE—adverse event, PE—premature ejaculation.

## 6. Comparative Effects of α1-Adrenoceptor Antagonists on Ejaculation and Sexual Function

Silodosin and tamsulosin are among the most selective antagonists of α1-adrenoceptors used in clinical practice. Silodosin shows particularly strong preference for the α1A subtype, demonstrating approximately 50-fold higher affinity compared with α1D and more than 160-fold higher affinity compared with α1B receptors (α1A > α1D > α1B) [53]. Its high selectivity results from favourable interactions within the α1A-adrenoceptor binding pocket, consistent with the typical binding mode of aminergic GPCR antagonists, in which Asp106 in transmembrane domain III plays a central role. Tamsulosin, a sulfonamide derivative, also displays high affinity for α1A and α1D receptors, with approximately ten-fold lower affinity for α1B (α1A ≈ α1D > α1B) [53,54]. Although less subtype-selective than silodosin, tamsulosin effectively blocks smooth muscle contraction in tissues relevant to male ejaculation. Both drugs have been reported to induce ejaculatory disturbances. The primary mechanism involves reduced seminal emission caused by impaired α1A-mediated contraction of the seminal vesicles, prostate, and vas deferens. This results in diminished propulsion of seminal fluid into the urethra and, in some cases, near-complete absence of antegrade ejaculation [49,55].

From a contraceptive perspective, a desirable α1-adrenoceptor antagonist should exhibit rapid onset of action, high α1A selectivity, predictable inhibition of seminal emission, and rapid reversibility following a single oral dose. Based on currently available pharmacological data, silodosin appears to best fulfil these characteristics, although dedicated comparative studies remain unavailable. Table 2 summarizes the pharmacokinetic and pharmacodynamic characteristics of the three most commonly used α1-adrenoceptor antagonists that may be considered for repurposing as non-hormonal male contraceptives. Among currently available agents, silodosin has the strongest prospective clinical evidence supporting further investigation as a potential non-hormonal male contraceptive, although direct comparative studies of contraceptive efficacy and optimal dosing remain unavailable [53]. Tamsulosin demonstrates intermediate suitability, whereas alfuzosin may be less suitable because of its lower α1A selectivity and weaker effects on ejaculatory function. Even so, direct comparative studies evaluating contraceptive efficacy, optimal timing of administration, and long-term safety in healthy reproductive-age men are still lacking.

Importantly, pharmacokinetic parameters should not be interpreted as direct measures of contraceptive pharmacodynamic protection. Although silodosin reaches peak plasma concentrations approximately 2.5 h after administration and has an elimination half-life of approximately 13 h, these parameters alone do not establish the duration of inhibition of seminal emission. The duration of contraceptive pharmacodynamic protection cannot be inferred from the plasma half-life of silodosin alone. The relationship between drug exposure, timing of administration, completeness of seminal emission suppression, and duration of the contraceptive effect remains to be established in dedicated pharmacokinetic–pharmacodynamic studies.

The impact of silodosin on male sexual function was evaluated by the Andrology Study Group of the Society of Urological Surgery—Turkey (SUST) (2020) [56] (Table 3). A total of 98 men aged 45–82 years completed the 3-month treatment period, during which they received silodosin 8 mg daily. After the first month, 46 participants reported anejaculation, and by the third month this number increased to 49. Despite drug-induced dry orgasm, improvements were observed in erectile function, including enhanced ability to achieve and maintain erections, increased frequency of successful erections, and overall improvement across erectile function domains. Benefits were also noted in men with premature ejaculation or suboptimal erection quality. Kobayashi et al. [57] further investigated the effects of silodosin on ejaculation in 15 healthy volunteers aged 26–47 years. Participants received either silodosin 4 mg or placebo twice daily for three days in a crossover design. Group A (n = 8) received silodosin first, followed by placebo, while Group B (n = 7) received the treatments in reverse order, with a minimum 1-week washout between phases. Silodosin induced anejaculation in all participants. Mean semen volume decreased from 2.9 mL at baseline to 0 mL, total sperm count from 276 × 10^6^ to 0, and fructose concentration dropped from 1461.5 mg/mL to undetectable levels. Semen parameters returned to baseline after three days without treatment. Importantly, no spermatozoa were detected in post-ejaculatory urine, indicating that silodosin did not induce retrograde ejaculation. Capogrosso et al. [58] studied the effects of silodosin 8 mg daily for 3 months in 100 men aged 30–88 years. Anejaculation occurred in 48 participants, and seven men discontinued therapy for this reason. Hypospermia, defined as a subjective reduction in ejaculate volume, was reported by 23 participants. Additional adverse effects included reduced orgasmic sensation (n = 11), absence of orgasmic sensation (n = 6), erectile dysfunction (n = 11), diminished sexual desire (n = 7), and feelings of reduced virility or masculinity (n = 5). In the study by Abdel-Kader et al. [59], 17 of 23 men treated with silodosin 8 mg/day experienced anejaculation. The treatment was administered until stone expulsion or for a maximum of four weeks. Similarly, the combination of silodosin 8 mg with mirabegron 50 mg resulted in anejaculation in 21 of 25 participants.

Tamsulosin may also contribute to ejaculatory dysfunction, although its impact is generally less pronounced than that of silodosin [55]. In a randomized, double-blind, placebo-controlled trial conducted by Kawabe et al. [60], the effects of silodosin 4 mg twice daily were compared with tamsulosin 0.2 mg once daily over a 12-week period. A total of 176 patients received silodosin (with one excluded from analysis) and 192 received tamsulosin. Abnormal ejaculation was the most frequently reported adverse effect in the silodosin group, occurring in 39 of 175 participants and leading to treatment discontinuation in five men. By contrast, tamsulosin resulted in abnormal ejaculation in only 3 of the 192 treated patients.

Kim et al. [61] investigated the impact of low-dose tamsulosin (0.2 mg/day) on sexual function. The incidence of de novo ejaculatory dysfunction was 10.2% after one month of therapy and 6.0% after three months. Low-dose tamsulosin did not significantly affect sexual function, as confirmed by the Danish Prostate Symptom Score (DAN-PSS), which showed no clinically meaningful changes (*p* > 0.05). These findings suggest that tamsulosin at this dose has a relatively limited effect on overall sexual function, although some participants reported ejaculatory dysfunction. The effect of tamsulosin 0.2 mg/day over 12 weeks was also evaluated by Song et al. [62] in a cohort of 177 men. No significant differences were observed between baseline and follow-up for erectile function, sexual satisfaction, ejaculatory function, or overall sexual activity. After 12 weeks, 13.4% of participants reported some form of ejaculatory dysfunction: 3.1% experienced delayed ejaculation, 3.9% anejaculation, 3.9% reduced ejaculatory force, 6.3% reduced ejaculatory volume, 7.1% diminished pleasure, 3.1% pain during ejaculation, and 2.4% reduced ejaculatory frequency. Chen et al. [63] treated 23 men with mild lower urinary tract symptoms and premature ejaculation using tamsulosin 0.4 mg/day for four weeks. The therapy was effective for premature ejaculation; however, approximately 30% of treated men developed ejaculatory dysfunction, while 13% reported orgasmic discomfort or anejaculation. In the study by Soliman et al. [64], men who had previously taken daily tamsulosin 0.4 mg/day—and in whom anejaculation (17 of 25) or reduced semen volume (8 of 25) was common—were offered an intermittent dosing regimen. Participants took the same dose every other day and were allowed to engage in intercourse on non-tamsulosin days. This treatment adjustment led to improved ejaculation in 20 of the 25 men.

Doxazosin is a long-acting, selective α1-adrenoceptor antagonist [65]. The drug has been shown to exert beneficial effects on sexual health in men with refractory erectile dysfunction [66]. In a study by Kirby et al. [67], doxazosin was administered for 52 weeks, beginning at 1 mg/day and titrated up to a maximum of 8 mg/day over approximately 10 weeks, depending on the International Prostate Symptom Score (IPSS) and maximum urinary flow rate (Qmax). Among the 275 men treated with doxazosin, only one reported abnormal ejaculation. Additionally, 10 participants experienced decreased libido, and 16 reported the onset of impotence. A parallel group of 264 men received the same doxazosin regimen in combination with finasteride 5 mg/day for 52 weeks. In this cohort, abnormal ejaculation occurred in 10 patients, impotence was reported by 30 men, and decreased libido by 6 participants.

Yamaguchi et al. [68] conducted a randomized study in which participants were assigned to two treatment groups: 53 men received silodosin 8 mg/day, whereas 44 men were treated with naftopidil 75 mg/day. After 12 weeks, anejaculation was reported by 45% of the 23 sexually active men in the silodosin group and by 8% of the 21 sexually active men in the naftopidil group. Ejaculatory dysfunction was therefore more frequent among men taking silodosin. Specifically, reductions in semen volume were reported by 87% of sexually active men receiving silodosin and 40% of those receiving naftopidil. Prolonged time to ejaculation occurred in 56% of the silodosin group and 33% of the naftopidil group, while diminished orgasmic sensation was reported by 50% and 39% of men, respectively. Sato et al. [69] evaluated the effects of naftopidil and silodosin in 26 men with premature ejaculation. Participants self-administered naftopidil 25 mg or silodosin 4 mg one hour prior to intercourse, using each medication at least three times. Silodosin was associated with a greater reduction in semen volume compared with naftopidil. Following silodosin administration, 13 of the 26 men experienced markedly reduced ejaculation or complete anejaculation. Overall, silodosin demonstrated greater efficacy on ejaculatory suppression than naftopidil in managing premature ejaculation.

Hisasue et al. [70] administered naftopidil (50 mg/day or 100 mg/day) or tamsulosin (0.2 mg/day or 0.4 mg/day) to 17 men in a crossover design. Participants were divided into two groups. Group A received the medications in the following sequence: tamsulosin 0.2 mg/day, tamsulosin 0.4 mg/day, naftopidil 50 mg/day, and naftopidil 100 mg/day, whereas Group B received the drugs in the reverse order. Each dose was taken for three days, followed by a seven-day washout period before the next treatment phase. The lowest semen volume was recorded after tamsulosin 0.4 mg (approximately 1.51 mL), followed by tamsulosin 0.2 mg (approximately 1.75 mL), naftopidil 50 mg/day (approximately 2.48 mL), with the highest volume observed after naftopidil 100 mg/day (approximately 2.7 mL). Baseline volume was 2.72 mL. Thus, ejaculate volume decreased noticeably in participants taking both doses of tamsulosin. Sperm concentration also followed a similar pattern. The lowest values were observed after tamsulosin 0.4 mg (approximately 30 × 10^6^/mL), followed by tamsulosin 0.2 mg (approximately 60 × 10^6^/mL), naftopidil 50 mg/day (approximately 61 × 10^6^/mL), and the highest values after naftopidil 100 mg/day (approximately 65 × 10^6^/mL). Baseline concentration was approximately 67 × 10^6^/mL. Total fructose concentration in the ejaculate—a marker of seminal vesicle function—was lowest after tamsulosin 0.4 mg (approximately 5 mg), then tamsulosin 0.2 mg (approximately 5.5 mg), naftopidil 50 mg/day (approximately 8 mg), and highest after naftopidil 100 mg/day (approximately 9.5 mg). Baseline concentration was approximately 10.5 mg. Importantly, no spermatozoa were detected in the urine of any participant, indicating that neither treatment induced retrograde ejaculation.

In the trial conducted by Akin et al. [71], 108 men with premature ejaculation were assigned to five groups, each receiving a different alpha-blocker. All medications were administered continuously for a minimum of 14 days. Silodosin (4 mg) was given to 21 men, tamsulosin hydrochloride (0.4 mg) to 23 men, alfuzosin (10 mg) to 22 men, terazosin (5 mg) to 21 men, and doxazosin mesylate (4 mg) to 21 men. Anejaculation occurred in 5 of the 21 men treated with silodosin and in three of the 23 men treated with tamsulosin hydrochloride; however, it was not associated with discomfort. No cases of anejaculation were reported in the groups receiving alfuzosin, terazosin, or doxazosin mesylate. Importantly, all alpha-blockers demonstrated efficacy in delaying ejaculation, with silodosin exhibiting the greatest effect.

In the study by Rosen and Fitzpatrick [72], 5999 men were screened for ejaculatory dysfunction using questionnaires, with 5873 providing analyzable responses. Compared with the control group, treatment with doxazosin or terazosin had no significant effect on ejaculatory function. Tamsulosin monotherapy, however, was associated with a higher incidence of anejaculation (52.3%), reduced semen volume, and decreased ability to ejaculate during sexual activity. Men treated with alfuzosin (10 mg) experienced significantly fewer symptoms of ejaculatory dysfunction compared with the control group. Additionally, the combination of an alpha1-blocker with a 5α-reductase inhibitor was associated with a higher frequency of dry ejaculation.

Hellstrom et al. [73] evaluated the effects of tamsulosin and alfuzosin on semen parameters in 48 healthy men. In a randomized, three-way, crossover, double-blind study, tamsulosin was associated with reduced semen volume, lower sperm concentration and motility, decreased normal morphology, and a lower proportion of men with normal semen viscosity. Seventeen participants experienced anejaculation on tamsulosin. In contrast, alfuzosin had minimal negative effects, slightly increasing semen volume, sperm concentration, total sperm count, and normal morphology, while preserving motility and semen viscosity. Fructose positivity was observed in all men receiving alfuzosin compared with 84% in the tamsulosin group.

In summary, silodosin and tamsulosin are selective α1-adrenoceptor antagonists, with silodosin exhibiting a particularly strong affinity for the α1A subtype. Both drugs can induce ejaculatory disturbances, primarily by reducing contraction of the seminal vesicles, prostate, and vas deferens, leading to decreased ejaculate volume or anejaculation. Silodosin has a more pronounced effect than tamsulosin, often causing complete anejaculation without inducing retrograde ejaculation, while also showing some benefits for erectile function and management of premature ejaculation. Tamsulosin may reduce semen volume and affect sperm parameters at higher doses, but low-dose therapy (0.2 mg/day) has minimal impact on sexual function. Alfuzosin and doxazosin have a milder effect on ejaculation, with alfuzosin even slightly improving semen parameters and doxazosin rarely causing ejaculatory dysfunction. Overall, among α1-blockers, silodosin carries the highest risk of ejaculatory side effects, tamsulosin has a moderate effect, and alfuzosin and doxazosin the lowest.

Safety data for α1-adrenoceptor antagonists have been generated predominantly in older men receiving long-term treatment for benign prostatic hyperplasia. Consequently, extrapolation of cardiovascular safety, tolerability and sexual side-effect profiles to healthy reproductive-age men requires caution. Younger men differ substantially with respect to cardiovascular risk, concomitant medication use and treatment expectations. Dedicated safety studies in healthy volunteers will therefore be essential before clinical implementation of this strategy.

Another important limitation is the heterogeneity of the currently available evidence. Existing studies differ considerably with respect to patient populations, underlying indications for treatment, study design, outcome measures, and methods used to assess ejaculatory function. Such heterogeneity limits direct comparison between studies and precludes robust conclusions regarding the true contraceptive potential of α1-adrenoceptor antagonists. Moreover, most clinical evidence originates from studies in which ejaculatory dysfunction was reported incidentally rather than prospectively investigated as a primary reproductive endpoint.

**Table 3:** α1-antagonist effects on male sexual function.

α1-Adrenergic Blocker	Dosage	Effect on Ejaculations	Effect on Other Sexual Functions	Ref.
Silodosin	8 mg per day for 3 months.	In the first month of therapy, 46 patients developed anejaculations, and 49 in the third month. Beneficial effects on premature ejaculation	After 3 months of treatment, men with drug-induced anejaculation reported improved ability to achieve and maintain erections.	Andrology Study Group of Society of Urologic Surgery—Turkey [74].
Silodosin	4 mg twice a day for 3 days.	Anejaculation in all men. Mean sperm volume—0 mL Mean total sperm count—276.0 × 10^6^ Mean fructose concentration was not available. No spermatozoa in the urine	Not reported.	Kobayashi et al. [57]
Silodosin	8 mg per day for 3 months.	Anejaculation in 48 of 100 men. 7 men discontinued due to anejaculation. Hypospermia in 23 of 100 men.	Of reduced orgasmic sensation in 11 of 100 men. Lack of orgasmic sensation in 6 of 100 men. Erectile dysfunction in 11 of 100 men. Low sexual desire/interest in 7 of 100 men. Feelings of reduced masculinity/masculinity in 5 of 100 men.	Capogrosso et al. [58].
Silodosin	0.8 mg per day, period of use up to the time of stone removal or for 4 weeks maximum.	Anejaculation in 17 of 23 men.	No information.	Abdel-Kader et al. [59]
Silodosin	8 mg per day for 12 weeks.	Anejaculation in 45% of the 23 sexually active patients A reduction in semen volume in 87% of the 23 sexually active patients An increase in time to ejaculation in 56% of the 23 sexually active patients	A decrease in orgasm in 50% of the 23 sexually active patients	Yamaguchi et al. [68].
Silodosin	4 mg on demand 1 h before sex	Semen volume reduction. 13 of 26 men had significantly decreased ejaculation or ‘no ejaculation’. Effective against premature ejaculation.	No information.	Sato et al. [69].
Silodosin	For at least 14 days at a dose of 4 mg.	Anejaculation in 5 of 21 men. Effective against premature ejaculation.	No information.	Akin et al. [71]
Silodosin	4 mg twice a day for 12 weeks.	Abnormal ejaculation in 39 of 175 men.	No information.	Kawabe et al. [60].
Tamsulosin	0.2 mg per day for 12 weeks.	Abnormal ejaculation in 3 of 192 men.	No information.	
Tamsulosin	0.2 mg per day for 3 months	Demonstrated de novo ejaculatory dysfunction incidence of 10.2% at 1 month and 6.0% at 3 months. Any adverse on ejaculatory function	no significant effect on sexual function.	Kim et al. [61].
Tamsulosin	0.2 mg per day for 12 weeks.	No overall effect on ejaculatory function was observed; however, 13.4% of men reported ejaculatory dysfunction, including delayed ejaculation (3.1%), anejaculation (3.9%), reduced ejaculatory strength (3.9%), reduced ejaculatory volume (6.3%), reduced pleasure (7.1%), pain during ejaculation (3.1%), and reduced ejaculatory frequency (2.4%).	No effect on erections, satisfaction or sexual activity.	Song et al. [62]
Tamsulosin	0.4 mg per day for 4 weeks.	Ejaculatory dysfunction in about 30% of men. 13% of men experienced orgasm and anejaculation discomfort.	No information.	Chen et al. [63]
Tamsulosin	0.4 mg daily alternate days for 3 months. Not taken on day of sexual activity	Ejaculation improvement in 20 out of 25 patients	No information.	Soliman et al. [64]
	0.4 mg per day	Anejaculation in 17 of 25 men. Reduced ejaculate volume in 8 of 25 men.	No information.	
Tamsulosin	0.2 mg per day for 3 days.	1.75 mL of semen volume. About 60 × 10^6^ of sperm concentration. About 5.5 mg of total fructose level. No sperm in urine.	No information.	Hisasue et al. [70]
Tamsulosin	0.4 mg per day for 3 days.	1.51 mL of semen volume. About 30 × 10^6^ of sperm concentration. About 5 mg of total fructose level. No sperm in urine.	No information.	
Tamsulosin	For at least 14 days at a dose of 0.4 mg.	Anejaculation in 3 of 23 men. Effective against premature ejaculation.	No information.	Akin et al. [71]
Tamsulosin	No information.	More frequent anejaculation (52.3%). Decreased semen volume. Less capacity to ejaculate during sexual activity. Increased anejaculation risk.	No information.	Rosen et al. [72]
Tamsulosin	0.8 mg daily for 5 days.	Anejaculation in 17 of 48 men. A decrease in the volume of semen by an average of 2.4 mL. Mean semen sperm concentration—3.1 million/mL. A decreased mean sperm count of 54.6 million. In 64.5% of men, normal sperm viscosity. Motile sperm decreased by 13.8%. An increase in the percentage of sperm with abnormal morphology of 0.6% The percentage of patients with a positive semen test for fructose was 84%.	No information.	Hellstrom & Sikka [73]
Tamsulosin	0.4 mg 4 times daily for 12 weeks.	Abnormal ejaculation in 4 of 83 men. Retrograde ejaculation in 1 of 83 men 71.33% of participants report ‘little difficulty’ or ‘no difficulty’ with ejaculation	At baseline IIEF = 17.76 ± 9.20 (n = 82), at week 12 IIEF = 19.81 ± 9.28 (n = 80)	Pompeo et al. [74]

## 7. Regulatory Pathways for New Therapeutic Indication of Authorised Medicinal Products

α1-Adrenoceptor antagonists are already authorised medicinal products; therefore, their development as male contraceptives would represent drug repurposing rather than the development of a novel active substance. This approach may offer important regulatory and developmental advantages because existing data on pharmaceutical quality, pharmacokinetics, pharmacological properties, nonclinical toxicology, and clinical safety can provide an established evidence base and may reduce the need to repeat selected elements of early-stage development. However, these data cannot substitute for the evidence required to establish a new contraceptive indication. In particular, clinical development would need to demonstrate reliable contraceptive effectiveness in the intended population, characterize the safety of repeated or on-demand administration in healthy reproductive-age men, and establish reversibility of the contraceptive effect.

In the United States, the regulatory framework governing medicinal products is primarily established by the Federal Food, Drug, and Cosmetic Act (FD&C Act), codified in Title 21 of the United States Code (21 U.S.C. § 355) [75], with post-approval changes further governed by the implementing regulations set out in Title 21 of the Code of Federal Regulations, § 314.70 [76]. Applications for a new therapeutic indication for approved medicinal products are generally submitted as supplemental New Drug Applications (sNDAs). Alternatively, where appropriate, an applicant may seek approval through a 505(b)(2) application in accordance with 21 CFR § 314.54 [77], which permits partial reliance on existing safety and efficacy data pursuant to 21 U.S.C. § 355(b)(2) [75].

The regulatory framework governing medicinal products within the European Union is primarily established by Regulation (EC) No 726/2004, current consolidated version 28 January 2022 [78] and Directive 2001/83/EC consolidated version 1 January 2025 [79]. Subsequent post-authorisation modifications are governed by Commission Regulation (EC) No. 1234/2008, current consolidated version 1 January 2025 [80]. Pursuant to that Regulation, the addition of a new therapeutic indication is generally classified as a major Type II variation [81]. This regulatory pathway enables the assessment and approval of new therapeutic indications within the existing marketing authorisation. EMA guidance defines Type II variations as major changes that may have a significant impact on the quality, safety or efficacy of a medicinal product, and specifically addresses applications involving new or modified therapeutic indications [80,81]. The precise regulatory pathway would depend on the existing marketing authorisation, the proposed indication, the formulation and dosing strategy, and the evidence available to the applicant.

Thus, repurposing may shorten development by leveraging established pharmaceutical, pharmacological, toxicological, pharmacokinetic, and clinical safety information, but it should not be interpreted as permitting a direct transition from an approved urological indication to a contraceptive indication. The main potential advantage is that existing evidence may reduce the need to repeat selected elements of early-stage development and allow development resources to be concentrated on the evidence that remains specific to the new indication, particularly clinical studies of contraceptive effectiveness, safety in the intended population, acceptability, dosing, and reversibility. The extent of any reduction in development time and cost would depend on the existing evidence package, the proposed formulation and dosing regimen, and regulatory requirements for the new indication. Accordingly, repurposing has the potential to reduce both development time and cost relative to de novo drug development, although the magnitude of these savings cannot be reliably estimated without a product-specific development plan and regulatory assessment.

## 8. Discussion

Within the framework of an emission-blockade strategy, profound suppression of sperm delivery is deemed a vital pharmacodynamic objective; however, it remains insufficient to establish clinical contraceptive effectiveness on its own. In particular, a statistically significant reduction in mean sperm count or semen volume does not establish reliable protection against pregnancy, because residual sperm delivery may remain sufficient for conception. Similarly, anejaculation or a negative post-ejaculatory urine test should not be assumed to constitute a validated surrogate endpoint for pregnancy prevention. Accordingly, clinical development of α1A-adrenoceptor antagonists as male contraceptives should distinguish between pharmacodynamic endpoints—such as completeness of seminal emission suppression, sperm count per ejaculate, and semen volume—and the clinical endpoint of pregnancy prevention. The prospective study by Nouh et al. [48] provides important evidence linking marked suppression of sperm output with a lower observed pregnancy incidence, but the available evidence remains insufficient to establish the reliability of contraception for individual sexual exposures.

Male contraceptive development faces unique scientific and ethical challenges. Hormonal male contraceptive methods generally exert their contraceptive effect by suppressing the hypothalamic–pituitary–gonadal axis, thereby reducing intratesticular testosterone and spermatogenesis. In contrast, α1-adrenoceptor antagonists are intended to interfere with sperm transport and seminal emission without directly suppressing spermatogenesis. Novel technologies, particularly those with germline-modifying potential, raise additional ethical considerations, especially regarding unintended pregnancies and possible impacts on male fetuses.

Acceptability is particularly relevant for an emission-blockade contraceptive because the intended pharmacological effect may be perceived by users as a change in ejaculation. Kaur et al. [10] addressed this issue in a large multinational discrete-choice study of potential male contraceptive users and their female partners. The study explicitly included ejaculation-related attributes, ranging from no change to reduced or absent ejaculate, alongside administration route, dosing frequency, onset, reversibility, and contraceptive efficacy. Administration format and timing of use were the strongest determinants of men’s product preferences, whereas changes in ejaculation had a substantially smaller influence on decision-making [10]. These findings suggest that changes in ejaculation do not necessarily preclude interest in a male contraceptive; however, they should not be interpreted as evidence that silodosin-induced anejaculation is clinically acceptable. Direct acceptability studies in men actually receiving an emission-blocking contraceptive and in their partners remain necessary.

Market considerations further complicate development. Contraceptives are preventative products used by healthy individuals, requiring exceptionally high safety and efficacy standards. Users expect both high contraceptive efficacy and excellent tolerability, and discontinuation is often driven by side effects or dissatisfaction. Globally, male-directed methods account for approximately 14% of contraceptive use, with higher prevalence in developed regions, yet no reversible male methods have reached the market since the condom’s introduction over 300 years ago [82]. Limited funding, high development costs, and stringent regulatory expectations have contributed to stagnation in male contraceptive innovation, with pharmaceutical companies largely withdrawing support despite persistent public health need [83].

Despite encouraging mechanistic and clinical findings, several important challenges remain before α1-adrenoceptor antagonists can be considered viable non-hormonal male contraceptives. Most available clinical data originate from studies conducted in older men receiving treatment for benign prostatic hyperplasia or lower urinary tract symptoms rather than healthy men of reproductive age. In these investigations, ejaculatory dysfunction was recorded as an adverse event rather than a predefined contraceptive outcome. Furthermore, pregnancy prevention, contraceptive reliability and partner-related outcomes have rarely been evaluated. Although the currently available evidence has progressed beyond proof-of-mechanism and now includes early randomized clinical evidence, it remains insufficient to establish reliable contraceptive efficacy. Even though selective α1A-adrenoceptor antagonists clearly impair seminal emission and reduce sperm transport into the urethra, reliable contraceptive efficacy has not yet been established. The recently published randomized placebo-controlled trial demonstrated significant reductions in semen volume, total sperm count per ejaculate, and pregnancy rate compared with placebo following daily silodosin administration. Yet, these findings require independent confirmation before they can be generalized to routine clinical practice [48]. The relationship between incomplete seminal emission, residual sperm delivery, and the actual probability of pregnancy has not yet been established. Dedicated prospective studies specifically designed to evaluate reproductive outcomes remain limited, although recent randomized clinical evidence has substantially expanded the available data.

Another important limitation concerns the lack of long-term safety data in healthy men. While α1-adrenoceptor antagonists have been used safely for many years in the treatment of LUTS/BPH, their chronic or repeated on-demand administration in young, healthy individuals has not been systematically evaluated. Particular attention should be paid to cardiovascular safety, treatment adherence, acceptability among users and their partners, and the potential influence of repeated dosing on sexual satisfaction and quality of life. The pharmacokinetic profile of candidate drugs also deserves further investigation. An ideal on-demand male contraceptive should combine rapid absorption, high α1A-adrenoceptor selectivity, predictable suppression of seminal emission, minimal systemic adverse effects, and rapid reversibility following discontinuation. Although silodosin currently appears to fulfil many of these characteristics, recent prospective clinical studies further support its potential as the leading candidate among currently available α1-adrenoceptor antagonists. Finally, future clinical development should focus not only on demonstrating biological activity but also on establishing clinically meaningful reproductive endpoints. Prospective randomized trials incorporating pregnancy prevention, reversibility of contraceptive effect, semen analysis, treatment acceptability, and partner-reported outcomes will be essential to determine whether α1-adrenoceptor antagonists can ultimately become a clinically applicable non-hormonal contraceptive strategy for men. These limitations do not undermine the biological rationale of α1-adrenoceptor blockade but highlight the gap between proof-of-concept and demonstrated contraceptive efficacy.

Recent advances in hormonal male contraception demonstrate plausible efficacy, often surpassing condoms, and may provide additional health benefits analogous to female oral contraceptives. Novel delivery systems, including transdermal, oral, and long-acting injectable androgens, may improve acceptability and adherence. The NES/T transdermal gel currently represents the most advanced hormonal male contraceptive candidate and provides an important benchmark against which non-hormonal strategies may ultimately be compared [84]. However, withdrawal of major industry sponsors highlights the necessity for government and nonprofit involvement to support long-term clinical development. Given the persistent global demand for additional contraceptive options, continued investment in male contraceptive research is critical to expand choice, reduce unplanned pregnancies, and provide equitable reproductive responsibility for both men and women. Against this background, further development of non-hormonal approaches, including α1-adrenoceptor antagonists, may complement rather than replace hormonal strategies, thereby expanding future contraceptive options for men.

Recent pharmacological studies indicate that selective blockade of α1-adrenergic receptors may provide a viable nonhormonal approach to male contraception. Prazosin, a non-selective α1 antagonist, was evaluated in ten healthy men but did not significantly alter semen parameters or induce azoospermia, limiting its contraceptive potential [82]. In contrast, tamsulosin, a selective α1A antagonist, induced complete anejaculation at 0.8 mg in all participants while preserving libido and orgasm, and silodosin produced reversible azoospermia and reduced semen volume when administered 3 h prior to ejaculation, without causing retrograde ejaculation [57,83]. Collectively, these findings indicate that receptor selectivity and timing of administration are critical determinants of reversible sperm suppression, supporting selective α1A-adrenoceptor blockade as a promising pharmacological strategy for non-hormonal male contraception. The study by Bhat and Shastry [49] represented the first prospective clinical evaluation of an α1A-adrenoceptor antagonist as a potential contraceptive agent. More recently, these preliminary observations were reinforced by the first randomized placebo-controlled trial evaluating reproductive outcomes in healthy fertile men, further strengthening the clinical evidence supporting selective α1A-adrenoceptor blockade [48]. Importantly, this study represents the first randomized placebo-controlled evaluation of reproductive outcomes in healthy fertile men and therefore marks a significant milestone in the clinical translation of α1A-adrenoceptor antagonists from mechanistic hypothesis to prospective contraceptive research. Nevertheless, both studies require independent validation before definitive conclusions regarding clinical efficacy can be drawn.

The available clinical evidence also highlights a fundamental distinction between acute pharmacological blockade of seminal emission and sustained contraceptive protection. The Bhat and Shastry [49] study demonstrated that silodosin administered approximately 3 h before intercourse could produce reversible azoospermia in the studied participants, whereas the Nouh et al. [48] study demonstrated progressively greater suppression of sperm output during repeated treatment and a lower observed pregnancy incidence than placebo. These findings are complementary but cannot be directly combined to define an optimal dosing regimen. In particular, the progressive effect observed during repeated treatment should not be assumed to reflect pharmacological accumulation, and the pharmacokinetic half-life of silodosin cannot be used as a surrogate for the duration of contraceptive protection. The critical development objective is therefore to establish a quantitative exposure–response relationship linking dose, timing, drug exposure, completeness of seminal emission suppression, residual sperm delivery, and pregnancy risk.

## 9. Conclusions

α1-Adrenoceptor antagonists could potentially be developed using either an event-driven or a repeated-dosing strategy. The available clinical evidence does not yet establish which approach provides the most reliable balance between completeness of seminal emission suppression, duration of effect, adherence, safety, and acceptability. An event-driven strategy may improve treatment acceptability while minimizing chronic drug exposure. However, successful implementation depends on reliable timing of administration, well-characterized pharmacokinetics and a defined exposure–response relationship, and complete suppression of seminal emission following each dose. Comparative pharmacokinetic studies evaluating optimal timing of administration, duration of action and reversibility will be equally important for determining the clinical feasibility of an on-demand non-hormonal male contraceptive. Future research should focus on prospective contraceptive trials conducted in healthy reproductive-age men, with pregnancy prevention as the primary clinical efficacy endpoint and reliable suppression of sperm delivery, semen parameters, and seminal emission suppression as important pharmacodynamic endpoints. Equally important will be the evaluation of patient-centered outcomes that are particularly relevant for contraceptive development but have received little attention to date. These include treatment adherence, user and partner acceptability, spontaneity of sexual activity, optimal timing of drug administration relative to intercourse, and the impact of repeated on-demand dosing on quality of life. Addressing these aspects will be essential to determine not only biological efficacy but also the real-world feasibility of α1-adrenoceptor antagonists as a non-hormonal contraceptive option. Taken together, the currently available evidence supports the biological rationale of α1-adrenoceptor blockade and now provides early prospective clinical validation of this concept. Nevertheless, the available data should still be regarded as early proof-of-concept rather than definitive proof of contraceptive efficacy until confirmed by larger multicentre clinical trials.

## Figures and Tables

**Table 2:** Pharmacokinetic and pharmacodynamic characteristics of selected α1-adrenoceptor antagonists relevant to potential male contraceptive development.

Parameter		Silodosin	Tamsulosin	Alfuzosin
α1A-adrenoceptor selectivity *	α1A/α1B selectivity	~162-fold	~9.5–10-fold	non-subtype-selective
	α1A/α1D profile	~50–55-fold	approximately similar affinity for α1A/α1D	non-subtype-selective
Tmax **		~2.5 h	4–6 h	~8 h (extended-release formulation)
Elimination half-life (t½)		~13 h	9–15 h	~5 h
Onset of action		Rapid	Moderate	Slower
Effect on seminal emission		Marked inhibition	Moderate inhibition	Limited inhibition
Current clinical evidence for further development		Prospective clinical evidence	Extensive pharmacodynamic/ejaculatory evidence	Limited ejaculatory effect

* Selectivity ratios are assay-dependent and are therefore reported from a common receptor-binding dataset where available; values should not be interpreted as directly predicting clinical uroselectivity or contraceptive efficacy. ** Pharmacokinetic parameters are formulation- and dose-dependent and should therefore be interpreted in the context of the specific formulation evaluated.

## Data Availability

No new data were created or analyzed in this study. Data sharing is not applicable to this article.
